# Gastric outlet obstruction secondary to solid-pseudopapillary neoplasm of the pancreas in an eight year old child. Report of a case

**DOI:** 10.1186/s13000-016-0465-7

**Published:** 2016-01-20

**Authors:** Rick Bidassek, Herbert Spelter, Daniel Gödde, Hubert Zirngibl, Peter C. Ambe

**Affiliations:** Helios Klinikum Wuppertal, Department of Surgery II, Witten – Herdecke University, 42283 Wuppertal, Germany; Institute of Pathology and Molecular Pathology, Helios Klinikum Wuppertal, Witten – Herdecke University, Wuppertal, Germany

**Keywords:** Pancreas, Pancreatic tumor, Solid pseudopapillary neoplasm, Gastric outlet obstruction, Immunohistochemistry

## Abstract

**Background:**

Solid pseudopapillary neoplasm is a rare cystic tumor of the exocrine pancreas. Abdominal pain or discomfort is the most common symptom, usually in young females.

**Case presentation:**

Herein we report the case of an 8 - year old child presenting with symptoms of gastric outlet obstruction.

**Conclusion:**

A solid pseudopapillary neoplasm of the pancreatic caput was diagnosed and surgically removed.

## Background

Solid pseudopapillary neoplasm (SPN) is a rare cystic tumor of exocrine pancreas with a female predominance. Clinically, it presents as a mass in the pancreas with a wide range of symptoms. Surgical exploration is indicated to release the intra-abdominal extrusion and for histopathology. SPN to the best of our knowledge has so far not been reported in patients below 9 years of age. Besides, gastric outlet obstruction has not been reported as the first sign of SPN.

## Case presentation

**Fig. 1:**
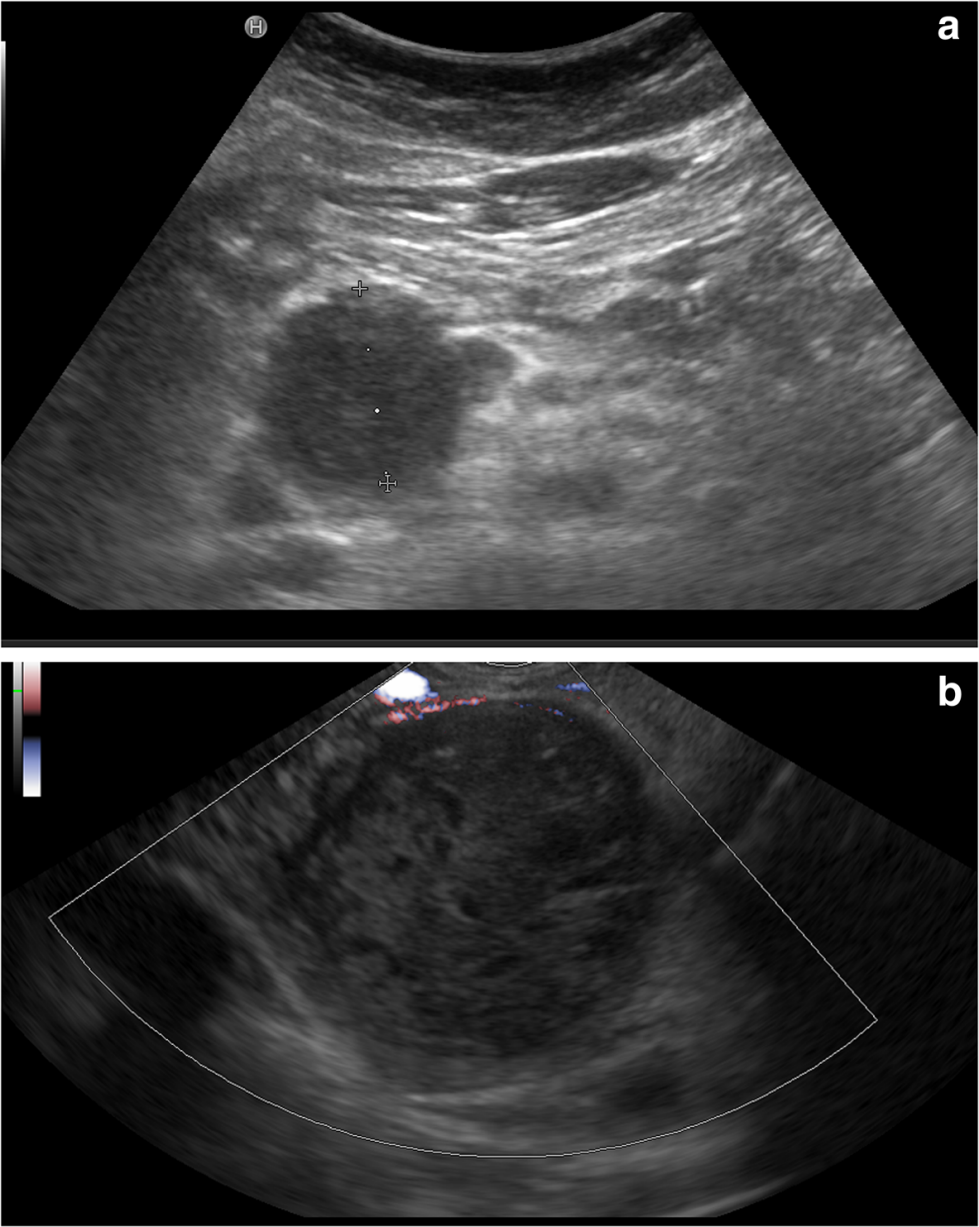
Findings from ultrasound sonography. **a** trans abdominal ultrasound sonography showing a mass in the pancreatic caput. **b** endoscopic sonography of the pancreas confirmed a large mass in the head of the pancreas

An 8-year old female patient presented in the pediatric unit with abdominal discomfort, nausea and bitter vomitus. Physical examination revealed a mass in the abdomen. Abdominal ultrasound sonography revealed a 3 cm large mass in the head of the pancreas. Endoscopic sonography confirmed a cystic tumor of the pancreatic caput and fine needle biopsies were taken for histopathology, Fig. 1a & b. Blood chemistry revealed elevated c-reactive protein (CRP: 7.2 mg/dl; normal range <0.5 mg/dl) and high white blood count (WBC: 16.2/nl; normal range: 4–10/nl). Serum lipase was mildly elevated (179 U/l; normal range: < 60 U/l). All liver enzymes including bilirubin were within normal range. Equally, the tumor markers carbohydrate antigen 19–9 (CA 19–9), carcinoembryonic antigen (CEA) and chromogranin A were within normal limits.

**Fig. 2:**
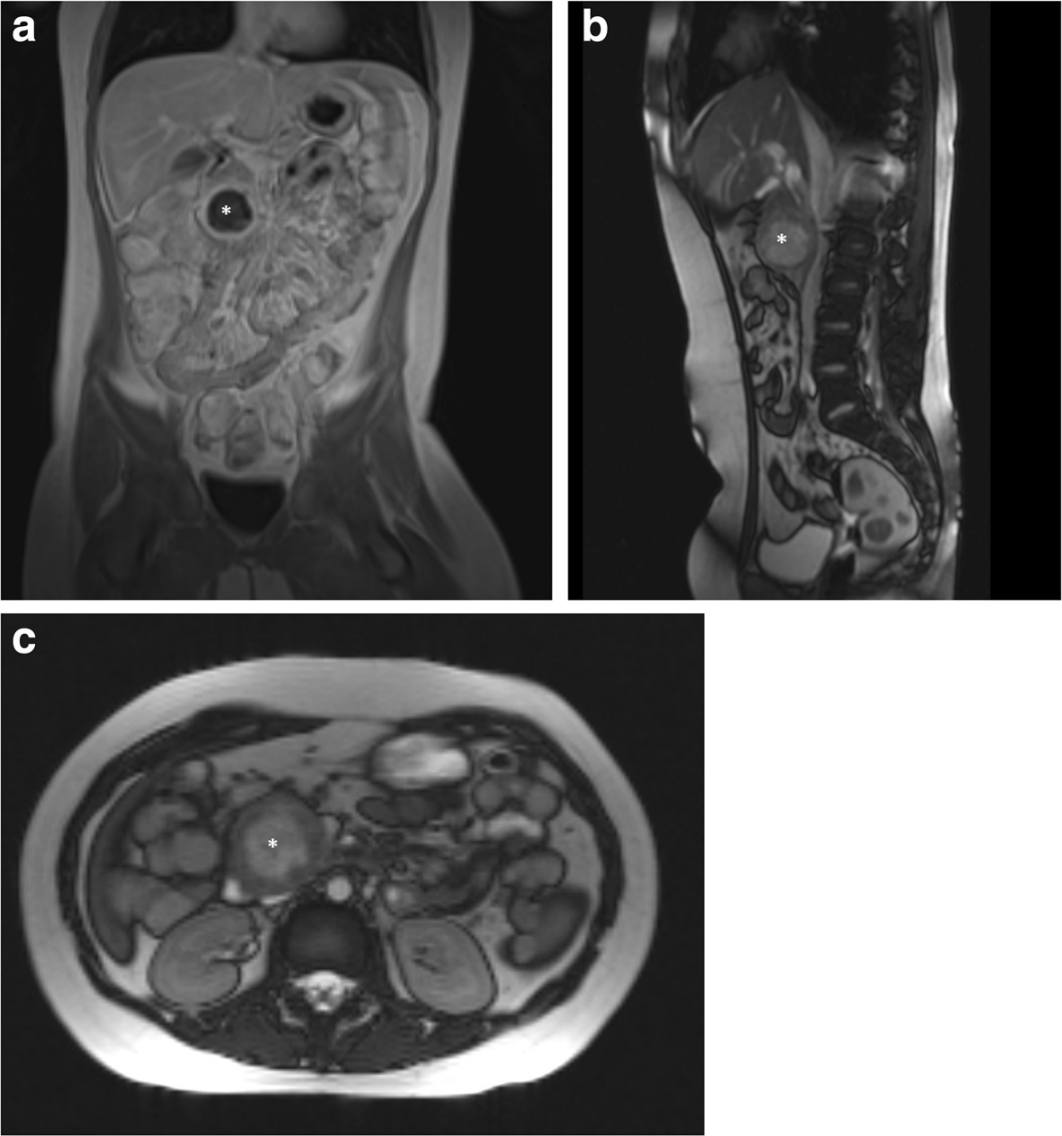
MRI scans in coronary. * SPN. **a** sagittal **b** tranversal **c** reconstruction

Magnet resonance imaging (MRI) of the abdomen showed a thick-walled, cystic mass in the head of the pancreas with compression of the duodenum, Fig. 2a–c. Histopathology from the fine needle biopsies was inconclusive. The case was discussed at the interdisciplinary board and surgical exploration was recommended.

**Fig. 3:**
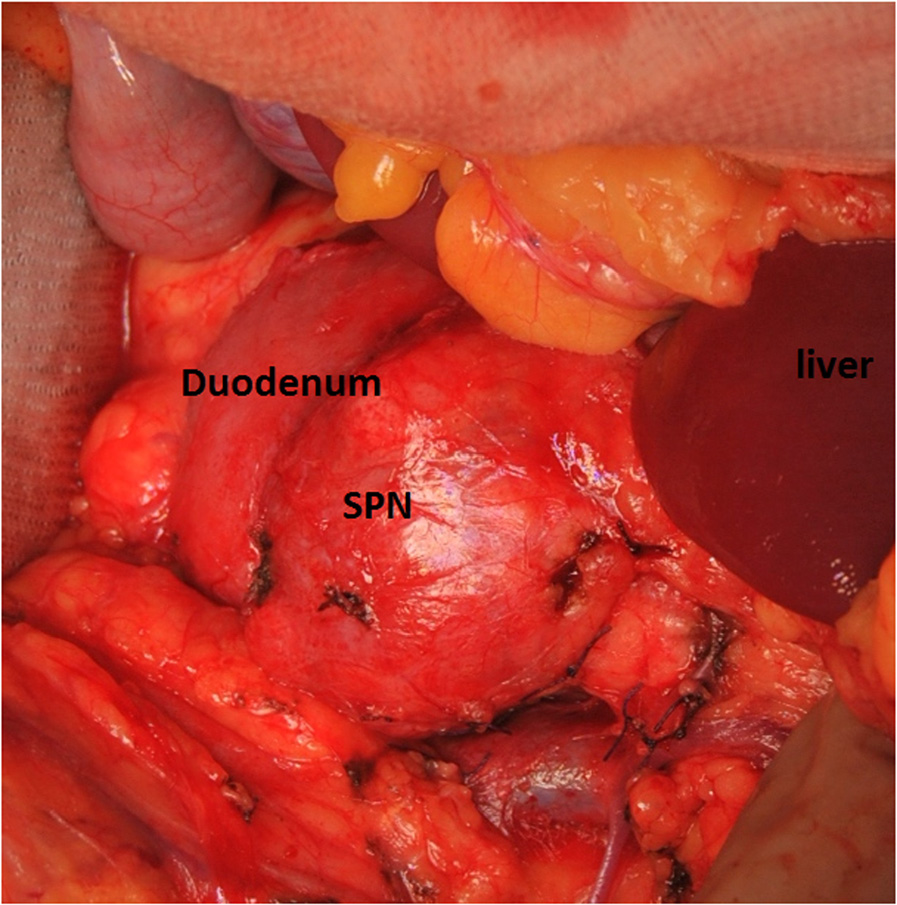
Intraoperative finding

A well capsulated mass of the pancreatic head without macroscopic signs of malignancy was found and completely enucleated during laparotomy, Fig. 3. Surgery and recovery were uneventful and the patient was discharged 7 days later with normal pancreatic function.

**Fig. 4:**
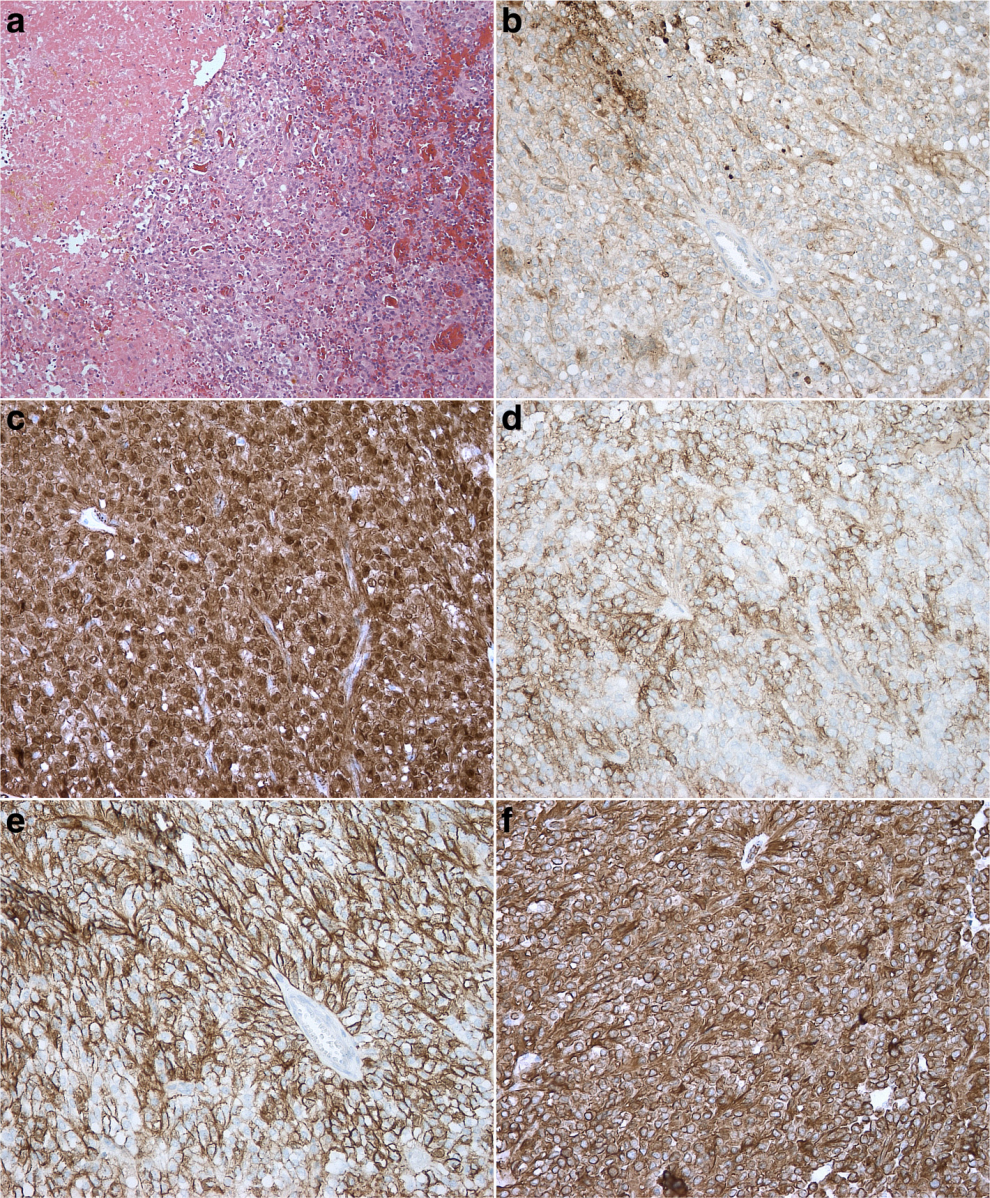
Histopathology and immunohistochemistry: **a** Hematoxylin and eosin. **b** Immunostaining for Antitrypsin. **c** Immunostaining for Beta-catenin. **d** Immunostaining for CD 10. **e** Immunostaining for CE 56. **f** Immunostaining for Vimentin

Histopathology confirmed the diagnosis of SPN following positive stains on immunohistochemistry with antitrypsin, vimentin, beta-catenin, CD 56 and CD 10, Fig. 4a–f.

## Discussion

SPN of the pancreas was first reported by Lichtenstein in 1934 [1]. In 1996 SPN was recognized by the World Health Organization as an independent histopathologic entity.

The incidence of SPN varies widely amongst reported series. Cubilla and Fitzerald [2] reported a 0.17 % incidence for SPN in a series of 645 pancreatic neoplasms. Morohoshi et al. [3] reported seven cases of SPN (2.7 %) after analyzing 264 pancreatic tumors. In yet another series of 336 cases, 1.48 % incidence of SPN was reported by Sheeham and colleagues [4]. Because all these incidences are based on adult or mixed population, the incidence of SPN in the pediatric population is estimated to be way below the 0.17 % reported by Cubilla and Fitzerald [2]. This notion is supported a report by Lack et al., who found just one case of SPN in a 30 - year review of data from the Boston Children’s Hospita [5].

The limited amount of publications on SNP is a reflection of the rare nature of this entity. In fact, a vast majority of the medical literature on this entity consists of case reports and small case series. The largest series so far is a review by Papavramidis et al. [6] including 718 cases. In this series, the prevalence of SPN was higher in female patients with a female to male ration of 10:1. The mean age at the time of diagnosis in this series was 22 years. Although an age range from 2 to 84 years was reported in their review, no single publication on SPN in a patient below the age of 9 years could be found in the English literature.

Although SPN can be clinically silent, patients might present with a mass in the upper abdomen with discomfort or pain [7]. Physical examination may reveal a mass in the mid abdomen. This is usually seen on ultrasound sonography. Endoscopic sonography with fine needle biopsy for histopathology might be helpful. The tumor is usually identified on MRI or computed tomography. Surgical exploration and resection is the mainstay of treatment [8, 9]. Surgical excision is directed towards symptom relief as well as to confirm the on histopathology and role out other malignant pancreatic lesions.

On like in other reports, signs of gastric outlet obstruction, i.e. mechanical obstruction of the upper gastro-intestinal tract with recurrent bitter vomitus in this case heralded SPN. Although no enzymatic analysis of the vomitus for bile of gastric enzymes was performed, the bitter nature of the vomitus suggested a proximal gastro-intestinal obstruction. This was confirmed by the identification of a large mass in the pancreatic head with duodenal compression (Fig. 3). A well-encapsulated mass of the pancreatic caput was completely enucleated during surgical exploration. Histopathology and immunohistochemistry confirmed the mass to be an SPN.

There are two distinctive findings in this case: the age at the time of presentation and the initial symptom. This to the best of our knowledge is the first reported case of SPN in a patient below nine years. The child in this case was eight years old at the time of diagnosis and gastric outlet obstruction has not been previously reported as an initial presentation of SPN.

## Conclusion

Taken together, this is the first reported case of SPN in a child below the age of nine presenting with bitter vomitus as a sign of gastric outlet obstruction. An interdisciplinary board should discuss all such cases. Surgical exploration with the goal of releasing symptoms is the main stay of treatment. Although SPN is mostly benign, there is a minimal risk of malignant transformation. Therefore, histopathology with immunohistochemical staining should be performed to confirm the diagnosis of SPN and role out malignant pancreatic tumors.

### Consent

Written informed consent was obtained from the patient´s legal representatives for publication of this Case Report and any accompanying images. A copy of the written consent is available for review by the Editor-in-Chief of this journal”.

## Abbreviations

CA: carbohydrate antigen
CEA: carcinoembryonic antigen
CRP: C-reactive protein
MRI: magnet resonance imaging
SPN: solid pseudopapillary neoplasm
WBC: white blood count
